# Supplementary material for: Otolith geochemistry reflects life histories of Pacific bluefin tuna
Source: PLoS One. 2022 Oct 14;17(10):e0275899. doi: 10.1371/journal.pone.0275899 (PMC9565424; doi:10.1371/journal.pone.0275899)
Supplement: S1 Fig — See Table 1 for details on collection. Asterisk (*) indicates visible crystal bond on surface of NZ02 that was removed from Fig 1. (PDF) [file pone.0275899.s002.pdf]

Otolith geochemistry reflects life histories of Pacific bluefin tuna

John A. Mohan[1,2], Heidi Dewar[3], Owyn E. Snodgrass[3], Nathan R. Miller[4], Yosuke Tanaka[5], Seiji Ohshimo[4,5], Jay R. Rooker[1], Malcom Francis[6], R. J. David Wells[1]

[1] Texas A&M University at Galveston, Department of Marine Biology, 1001 Texas Clipper Rd., Galveston, TX 77553, USA

[2] University of New England, School of Marine and Environmental Programs, 11 Hills Beach Rd, 04005

[3] Southwest Fisheries Science Center, National Marine Fisheries Service, 8901 La Jolla Shores Dr., La Jolla, CA 92037, USA

[4] The University of Texas at Austin, Jackson School of Geosciences, 2275 Speedway Stop C9000, Austin, TX 78712, USA

[5] Highly Migratory Resources Division, Fisheries Stock Assessment Center, Fisheries Resources Institute, Fisheries Research and Education Agency, 5-7-1, Orido, Shimizu, Shizuoka, 424-8633, Japan

[6] Pelagic Fish Resources Division, Fisheries Stock Assessment Center, Fisheries Resources Institute, Fisheries Research and Education Agency, 1551-8 Taira-machi, Nagasaki, 851-2213, Japan

[7] National Institute of Water and Atmospheric Research (NIWA), Private Bag 14901, Wellington 6021, New Zealand

Keywords: *Thunnus orientalis*, migration, biomineralization, trace elements, otolith chemistry

Supplemental Figure 1 (below). Elemental profiles of individual PBT otoliths collected from the Eastern Pacific (EPO), Western Pacific (WPO) and South Pacific (SPO); different colored lines represent individual fish from each region. See Table 1 for details on collection. Asterisk (*) indicates visible crystal bond on surface of NZ02 that was removed from Figure 1.

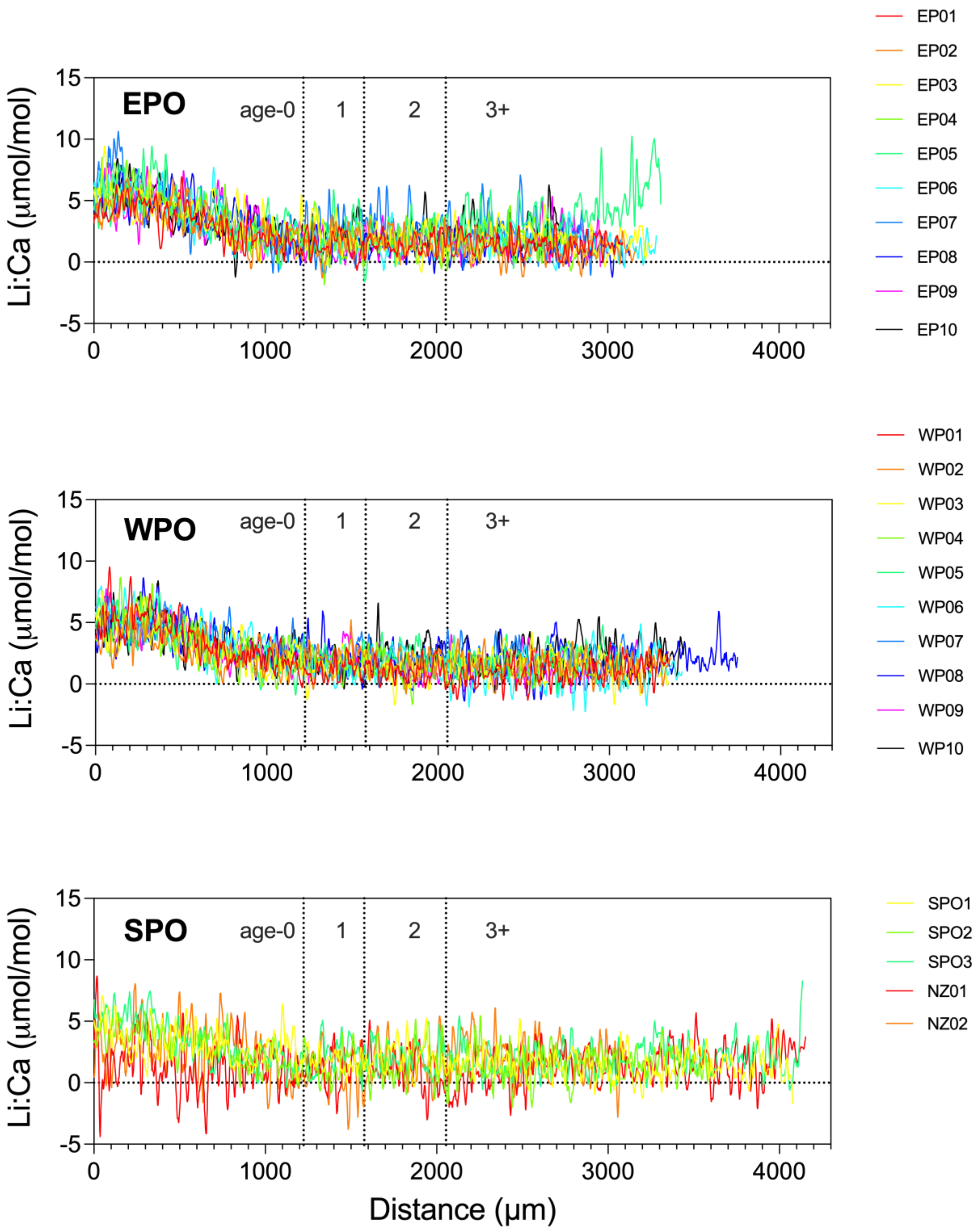
EPO
age-0
1
2
3+
Li:Ca (μmol/mol)
15
10
5
0
-5
0
1000
2000
3000
4000
EP01
EP02
EP03
EP04
EP05
EP06
EP07
EP08
EP09
EP10
WPO
WP01
WP02
WP03
WP04
WP05
WP06
WP07
WP08
WP09
WP10
SPO
SPO1
SPO2
SPO3
NZ01
NZ02
Distance (μm)

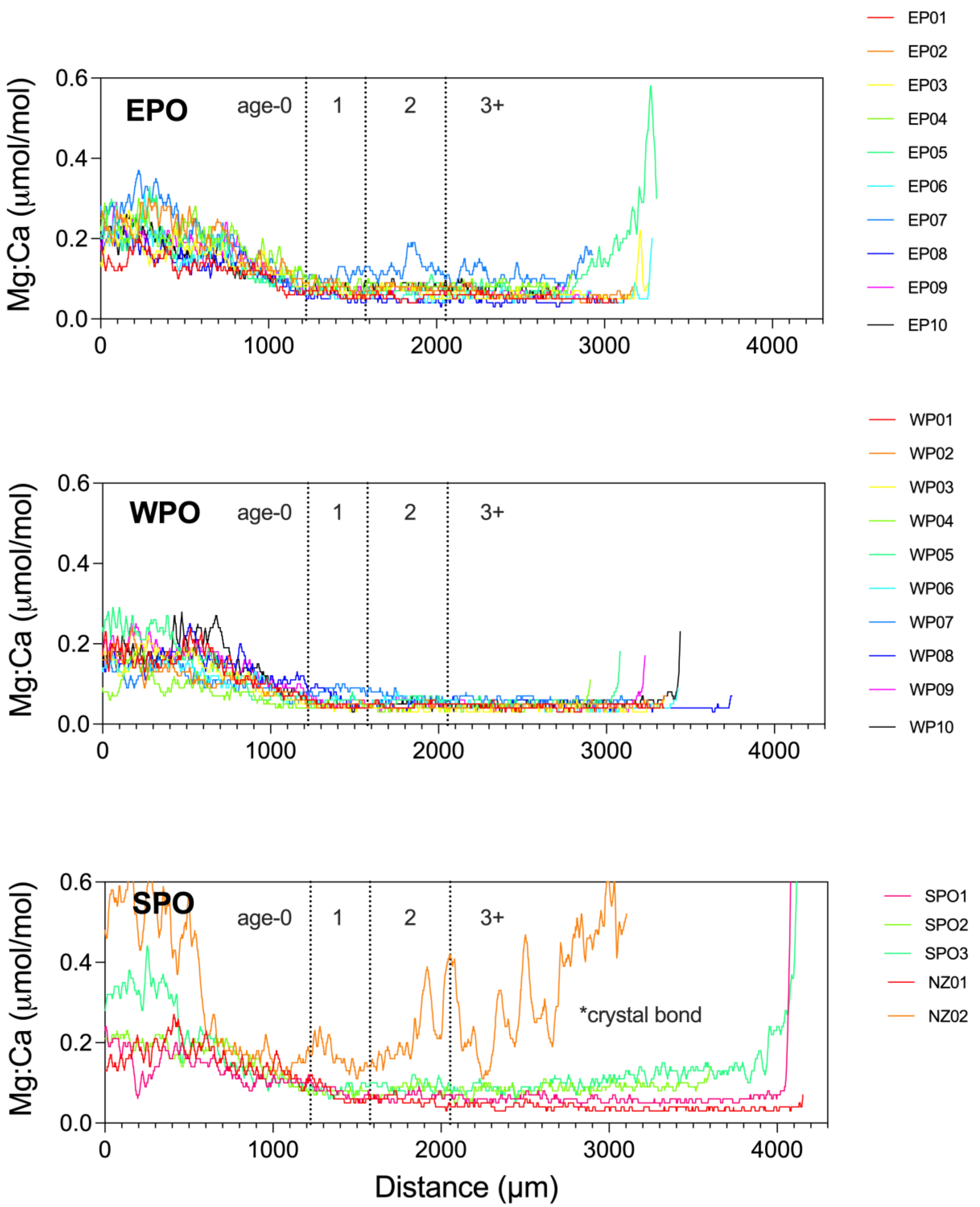
EPO
WPO
SPO
age-0
1
2
3+
Mg:Ca (µmol/mol)
0.0
0.2
0.4
0.6
0
1000
2000
3000
4000
Distance (µm)
*crystal bond
EP01
EP02
EP03
EP04
EP05
EP06
EP07
EP08
EP09
EP10
WP01
WP02
WP03
WP04
WP05
WP06
WP07
WP08
WP09
WP10
SPO1
SPO2
SPO3
NZ01
NZ02

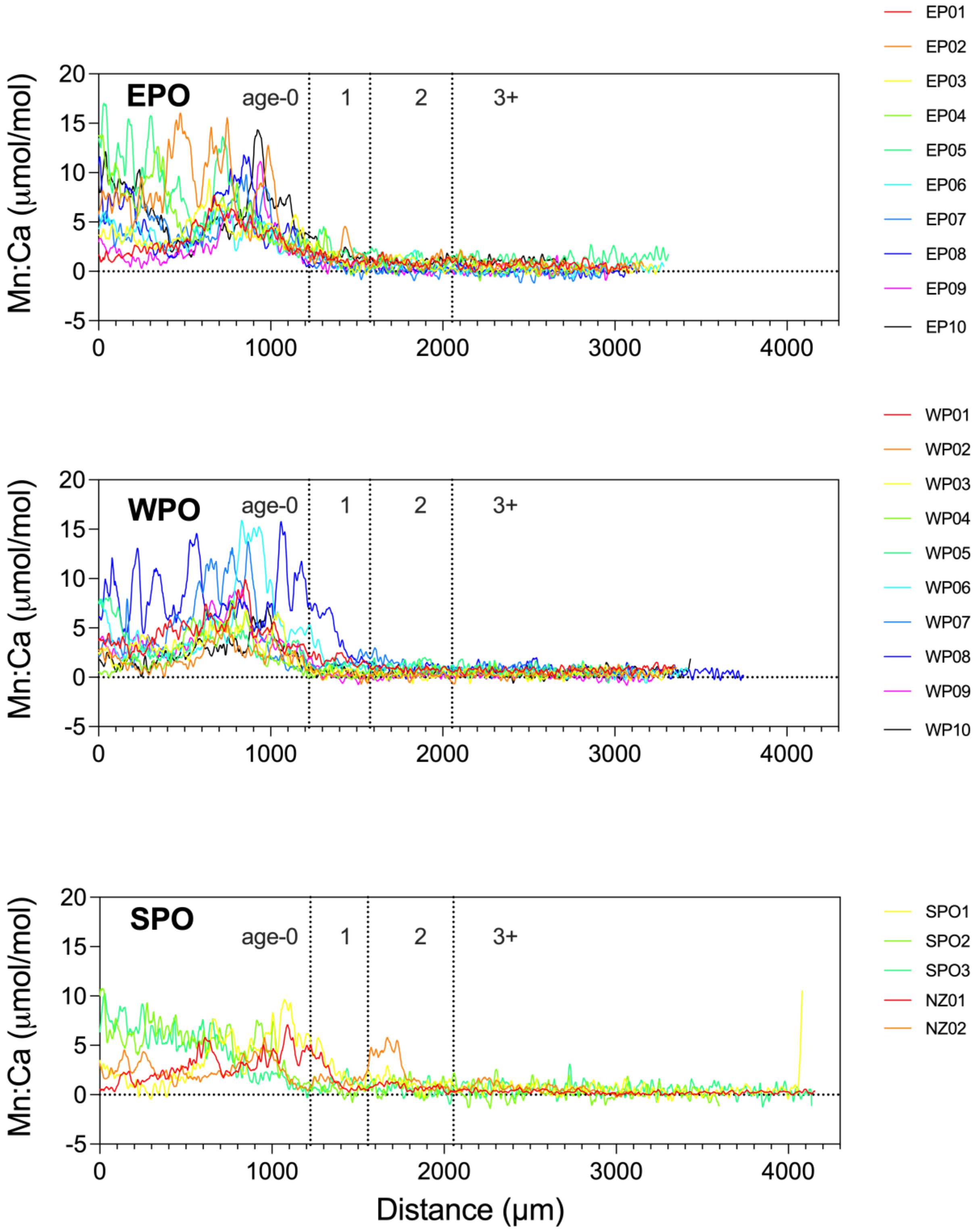
EPO
age-0
1
2
3+
Mn:Ca (µmol/mol)
EP01
EP02
EP03
EP04
EP05
EP06
EP07
EP08
EP09
EP10
WPO
WP01
WP02
WP03
WP04
WP05
WP06
WP07
WP08
WP09
WP10
SPO
SPO1
SPO2
SPO3
NZ01
NZ02
Distance (µm)

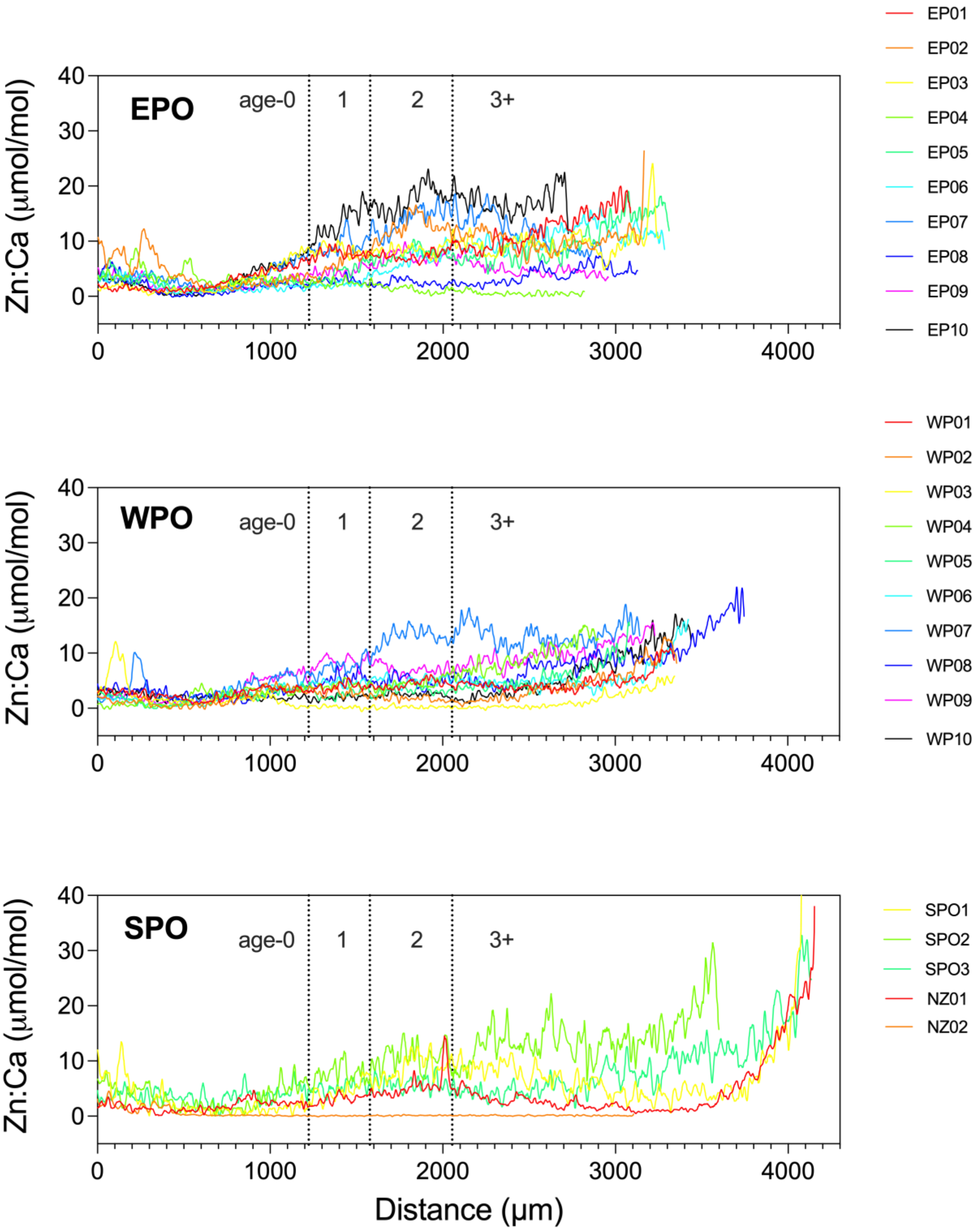
EPO
age-0
1
2
3+
Zn:Ca (µmol/mol)
EP01
EP02
EP03
EP04
EP05
EP06
EP07
EP08
EP09
EP10
WPO
WP01
WP02
WP03
WP04
WP05
WP06
WP07
WP08
WP09
WP10
SPO
SPO1
SPO2
SPO3
NZ01
NZ02
Distance (µm)

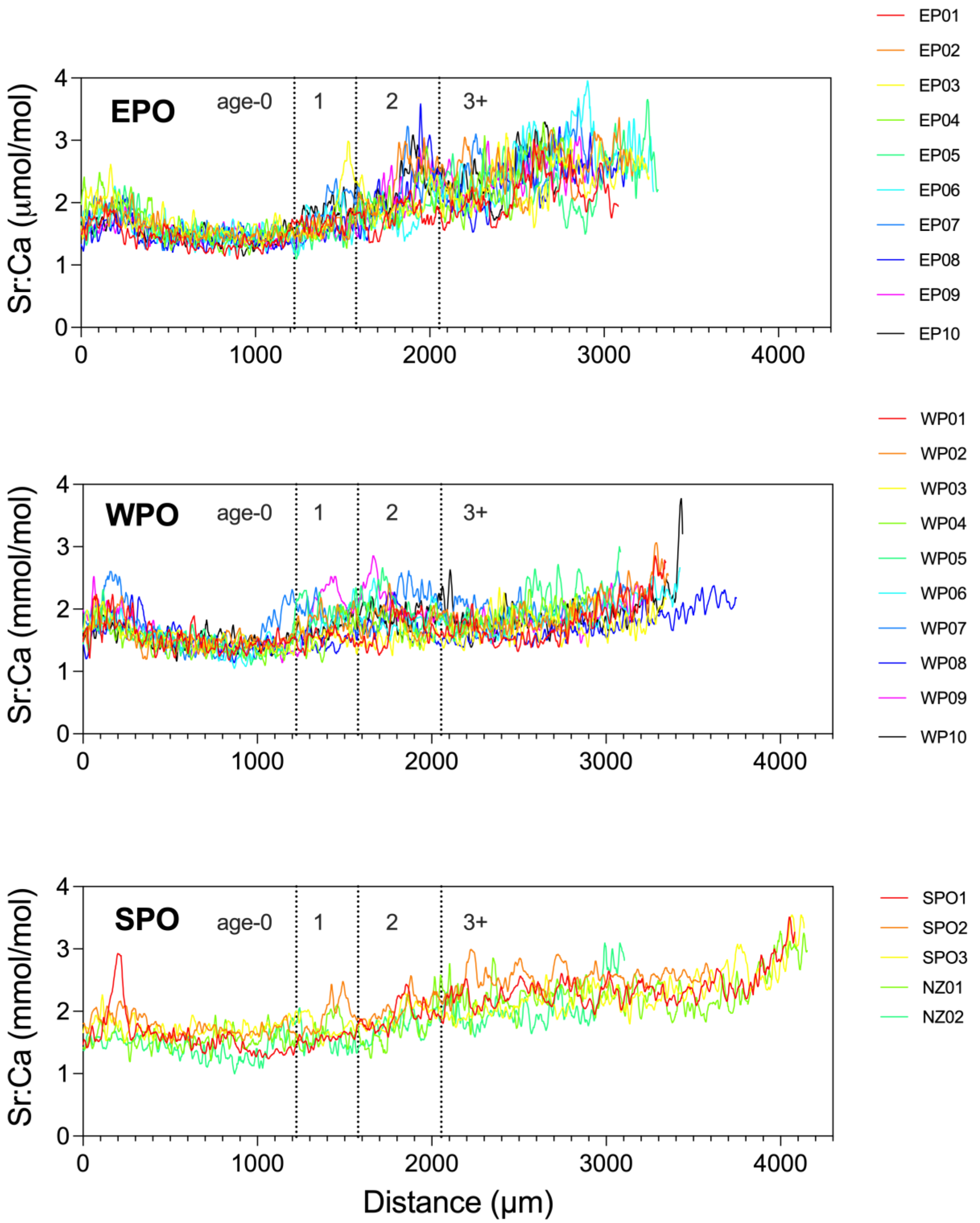
EPO
WPO
SPO
age-0
1
2
3+
Sr:Ca (μmol/mol)
Sr:Ca (mmol/mol)
Distance (μm)
0
1
2
3
4
0
1000
2000
3000
4000
EP01
EP02
EP03
EP04
EP05
EP06
EP07
EP08
EP09
EP10
WP01
WP02
WP03
WP04
WP05
WP06
WP07
WP08
WP09
WP10
SPO1
SPO2
SPO3
NZ01
NZ02

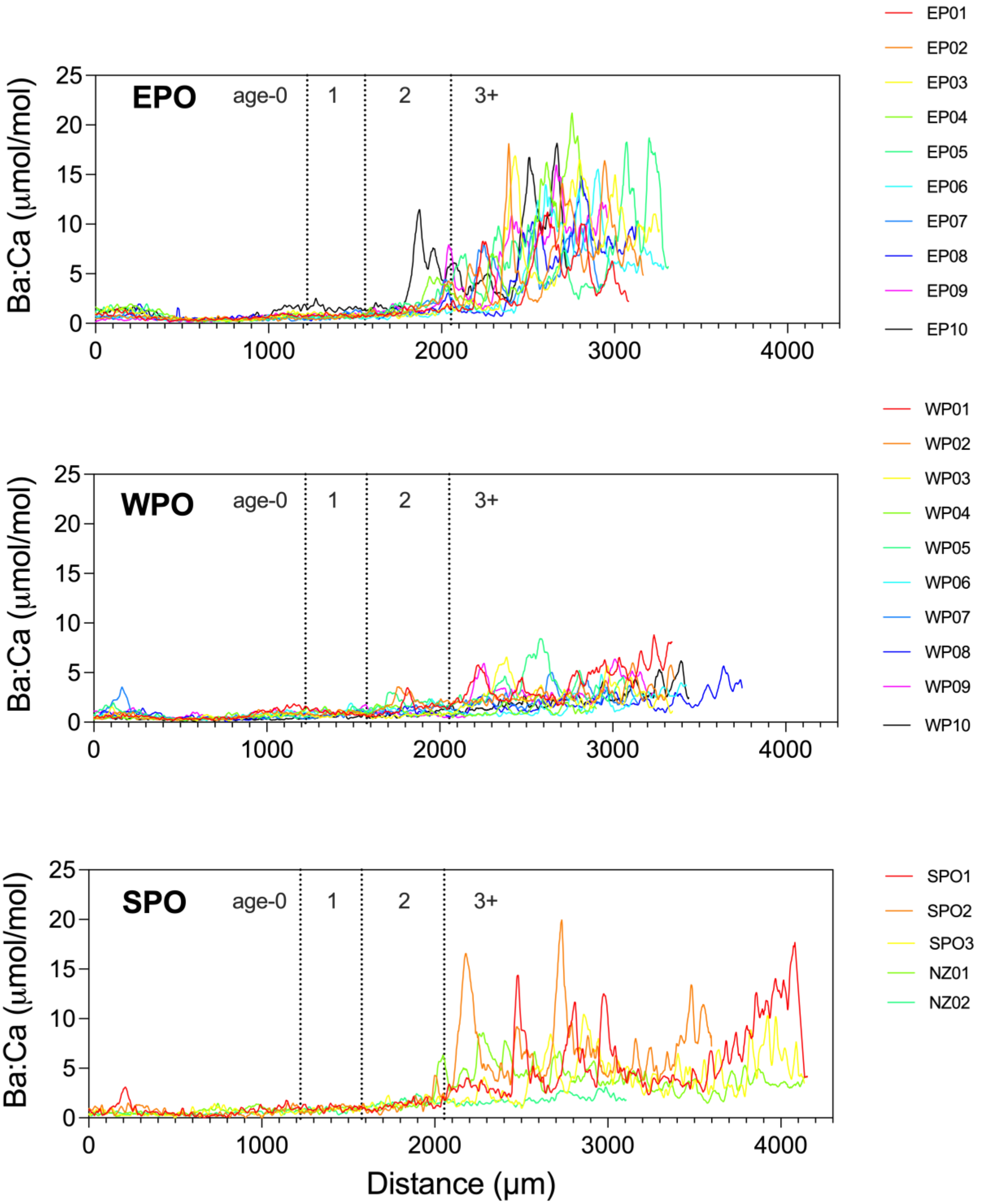
EPO
age-0
1
2
3+
Ba:Ca (μmol/mol)
0
5
10
15
20
25
0
1000
2000
3000
4000
EP01
EP02
EP03
EP04
EP05
EP06
EP07
EP08
EP09
EP10
WPO
WP01
WP02
WP03
WP04
WP05
WP06
WP07
WP08
WP09
WP10
SPO
SPO1
SPO2
SPO3
NZ01
NZ02
Distance (μm)
