# Supplementary material for: Otolith geochemistry reflects life histories of Pacific bluefin tuna
Source: PLoS One. 2022 Oct 14;17(10):e0275899. doi: 10.1371/journal.pone.0275899 (PMC9565424; doi:10.1371/journal.pone.0275899)
Supplement: Attachment — Submitted filename: Reviewers comments.docx [file pone.0275899.s003.docx]

This study presents an original research article on otolith trace element profile analysis of Pacific bluefin tuna (*Thunnus orientalis*), as an approach to provide records of their life history. Authors apply for the first time this technique in Pacific bluefin tuna collected across the species adult distributional range; covering distant areas from the Pacific Ocean (i.e., west, east, and south) and discuss on how different elements provide ontogenetic records of physiological and environmental histories.

Overall, the article is presented intelligibly and is easy to follow. Both experiments and statistics are performed to a high technical standard and are described in sufficient detail, so that other scientist can follow this study. Authors also state that data is available under request without restrictions. Results are rigorously reported and support the conclusions of the article. The motivation behind the work is clearly described, with straight and sound discussion of the observed results and in the limitations of the performed study.

I do believe that this research meets all criteria for publication in *PLOS ONE* journal. However, there are some minor issues that could be improved in my point of view before its publication as a high-quality manuscript:

- My biggest concern is with the readability of the figures and tables. I don't know if it is a problem with the system, or with the quality of the figures themselves, but they should be thoroughly revised as they are difficult to read as I see them. Please check them for quality standards and size format.
- I think the discussion lacks a little bit more, the microchemistry part is very well detailed and contextualized, but it seems to me a good opportunity to discuss a little bit more about migratory behaviours, contingents and discuss some implications of these findings for the Pacific bluefin tuna management and fisheries as well as and future research priorities. Besides, the introduction of the discussion needs to be refined a bit by looking at the connection between the ideas to be presented later.

If authors can address these minor issues, I do believe that this manuscript will be a great addition to the PLOS ONE articles collection and to the scientific community working with otolith or tuna management related studies. Bellow I have provided specific comments and/or questions to the authors that should be quite straightforward to address. I hope authors and editor can find these suggestions useful, and my congratulations for this nice piece of work, this is good science that needs to be shared!

**Introduction**

Line 85: I suggest using “core-to-edge” as it is more common in the literature and then in MS you are referring to that part as “edge”.

Lines 83-96: I would add also a small sentence saying that there is also substantial uncertainty on how intrinsic and extrinsic processes affect elemental incorporation into the otolith, and also that responses can be species specific (e.g. Hüssy et al., 2020; Sturrock et al., 2015).

Line 93: You can cite Farwell et al. 2001 at the end of this sentence if you want, which says “*The open ocean, pelagic environment of most scombrid species has made it challenging to conduct field research. Their high metabolic rates and specialized swimming result in significant space requirements if held in captivity. The major challenge is that tunas must swim continuously, which makes collecting and captive care difficult*”.

Farwell, C. J. (2001). 10. Tunas in captivity. *Fish physiology*, *19*, 391-412.

Line 99: And differentiate among contingents or fish with different life histories?

Line 108-109: I suggest replacing “LA ICP MS” by “LA-ICP-MS”

Line 109: From hatch time to adult?

Line 110: I feel that “comparing complete chemical profiles” would be more accurate than “comparing complete life histories”

Line 114: I suggest deleting “biominerals” at the end of the sentence.

**Material and Methods**

Lines 118-120: I think it will be nice to indicate here how many otoliths did you get from each region, and not later in Results section.

Line 120: I feel that a map would be a great added value here, where you can show the location of the 3 regions you are considering (instead of figure S1), and the location of the spawning areas. I think this will be a plus for the manuscript and will help the non PBT familiarized readers to locate the regions you mention throughout the manuscript (I needed to check in google maps to get the whole picture in my mind).

Table 1: I think that table 1 needs from some restructuration for the paper, for example, I am not sure if both PBT_ID and Otolith ID are relevant for the reader (perhaps choose only one). Same I am not sure if the fishing methods adds something here? Like this you could use the space you have left over to incorporate a “Region” column where you indicate if samples belonged to WPO, EPO or SPO a with the Location only, readers can be confused (as it was my case because I am not familiarized with the Pacific). Please add to the legend that size is fork length (FL). Also check that are some measures of FL and GG that are in bold.

Lines 129-130: “It was also impossible to know with certainty the migratory histories of sampled fish…” sure, but this is what you want to inspect no? I would suggest deleting this sentence and said only that fish were grouped by sampling region for comparisons.

Figure 1b: Perhaps it is worthy to write core, inflexion point and edge in the figure to help the readers locate the otolith position afterwards? Not sure…

Line 151. Crystalbond “adhesive” or “glue” instead of “cement”?

Line 165: Perhaps “Otolith sections and standards…”

Line 173: Perhaps you can cite Sturgeon et al., 2005 at the end of this sentence.

Sturgeon, R. E., Willie, S. N., Yang, L., Greenberg, R., Spatz, R. O., Chen, Z., ... & Thorrold, S. (2005). Certification of a fish otolith reference material in support of quality assurance for trace element analysis. *Journal of Analytical Atomic Spectrometry*, *20*(10), 1067-1071.

Line 190: Are these mean annual annuli? If so please state it in the text

Line 190: But this image only shows annual rings up to 3 years, how did you do for your older individuals?

Line 197: I know you mention later in the discussion, which is really nice, but I think it would also be worthy to mention here that time frame represented in each individual is different.

Line 200-201: Was your data normally distributed and present homogeneity of variances so that it justifies the use of one way ANOVA? If so please state, if not, consider using another non-parametric test. I see that you mention latter that a Brown- Forsythe test was performed and confirmed that variances of the populations from which the samples are drawn are equal, but what about normal distribution?

**Results**

Lines 212-216: I am not sure whether this should go here or in M&M, I feel that it does not fit in results, and it fits better in M&M sampling description.

Line 219: “...but it is possible that PBT…” ?

Line 236: Perhaps add “ratios” after “element:Ca”

Figure 2: “solid line represent mean values and shading depicts ± standard deviation across laser distance” I cannot see this in the figure I get. Please check just in case. “dashed vertical lines denote approximate annuli distances for the first 3 years of life…”, I think it will also be beneficial to add where the inflection point will be represented in the transect, as then you mention this in you discussion.

Figure 3: I personally found confusing the fact that horizontal barckets join regions that are not statistically significant, when usually it is the other way around, I would just mark between which there are significant differences.

Table 2: This table need a little bit of restructuring and editing. Please also indicate in the legend that significant P values are highlighted in bold in the table. Also add that inspected regional differences are between WPO, EPO and SPO.

Lines 282-283: I suggest expanding here and also to mention that although classification differences are more or less clear for WPO and EPO there is also some overlap. Also, that SPO is disperse, and not clear grouping can be observed. So, there are 2 SPO with similar values among them, 1 more “EPO like” and 2 more “WPO like”, as you can see in the classification of table 3.

Table 3: I recommend replacing “origin” by “capture” here. Perhaps you can highlight in bold the correct classifications of the table.

**Discussion**

Lines 296-297: I feel that this sentence is a bit weak as it is, and you should contextualise it more.

Lines 295-307: I think that some cohesion in the first paragraph of the discussion is missing.

Line 303: All of your fish are originated from the WPO no? not only the juveniles

Line 318: I suggest replacing “core-to-rim” by “core-to-edge”

Lines 318-322: Are all of these in the Pacific? Can you specify more on what similarities did you found? (e.g., enrichment/depletion for early life signatures etc.

Lines 322-324: And also continuous transects no?

Lines 325-333: I think you could also discuss here on the importance of the placement of the transect, as some elements do not precipitate equally in the otolith growth axis (you can check Artetxe-Arrate et al. 2021)

Artetxe-Arrate, I., Fraile, I., Clear, N., Darnaude, A. M., Dettman, D. L., Pécheyran, C., ... & Murua, H. (2021). Discrimination of yellowfin tuna Thunnus albacares between nursery areas in the Indian Ocean using otolith chemistry. *Marine Ecology Progress Series*, *673*, 165-181.

Line 365: What individual variability do you see in you fish for early life period of these elements as you are accounting for fish that were born at different years? Do you see some fish with signatures more similar to the East China Sea and other to Sea of Japan reported in Wells et al. 2020? Maybe you can discuss a bit more here

Line 382: Are lower temperatures expected for the WPO than for EPO and SPO? If so please state it

Line 402-404: But are these occasional dives long enough to be recorded in the chemical signature? I don’t think so…

Lines 419-424: We also found that otolith borders were enriched in Zn in comparison with the rest of the otolith for few different tuna species, perhaps because of Crystalbond inclusion, or because the otolith crystal composition of the border. I don’t know if it is worthy to also consider this in small sentence here?

I do miss a little discussion on contingents and migratory behaviour, and also to recap the importance of this knowledge for management (following your intro)

**Conclusion**

Perhaps recap your objectives and how you answer them with this study

Lines 448-449: Perhaps you can add “although it is often difficult to discern between the two”

Lines 461: And in other fish in general, as these type studies are mostly lacking for any species.

Best luck.
